# Letrozole Supplementation and the Increased Risk of Elevated Progesterone Levels on Trigger Day

**DOI:** 10.3389/fendo.2022.904089

**Published:** 2022-07-25

**Authors:** Rongju Liu, Liling Zhou, Xuemei Chen, Hongmei He, Zhaowei Cai

**Affiliations:** ^1^ Reproductive Medical Center, Dongguan Songshan Lake (SSL) Central Hospital, Dongguan City, China; ^2^ The Second School of Clinical Medicine, Southern Medical University, Guangzhou City, China

**Keywords:** IVF/ICSI, ovarian stimulation, letrozole, progesterone, trigger day

## Abstract

Although using letrozole (LE) during *in vitro* fertilisation and intracytoplasmic sperm injection (IVF/ICSI) has many advantages, it remains unclear whether LE induces an increase in progestogen during the late follicular phase. The objective of this study was to investigate whether progesterone levels increased under antagonist protocols supplemented with LE on the trigger day using a retrospective cohort study. The study included 1,133 women who underwent IVF/ICSI cycles from January 2018 to June 2020. After propensity score matching (PSM) for baseline characteristics, 266 patients with gonadotropin-releasing hormone-antagonist (GnRH-ant) were matched to 266 patients with letrozole + GnRH-ant (LE GnRH-ant) (PSM 1 cohort), and 283 patients with gonadotropin-releasing hormone-agonist (GnRH-a) were matched to 283 patients with LE GnRH-ant (PSM 2 cohort). In the PSM 1 cohort, patients in the LE GnRH-a group presented higher progesterone levels (1.22 ± 0.95 ng/mL vs 0.86 ± 0.60 ng/mL, *P* < 0.001), with a higher proportion of patients with progesterone level > 1.5 ng/mL (24.81% vs 7.52%, *P* < 0.001). In PSM 2 cohort, patients in the LE GnRH-a group presented higher progesterone levels on trigger day (1.23 ± 0.91 ng/mL vs 0.98 ± 0.61 ng/mL, *P* < 0.001), with a higher proportion of patients with progesterone level > 1.5 ng/mL (25.45% vs 12.70%, *P* < 0.001). In the PSM 1 cohort, progesterone levels on the trigger day increased by 0.05 ng/mL, with an increase in every retrieved oocyte in the LE GnRH-ant group (β 0.05 ng/mL [95% CI 0.04, 0.06], *P* < 0.001), whereas an increase of 0.02 ng/mL was observed in the GnRH-ant group (β 0.02 ng/mL [95% CI 0.01, 0.03], *P* < 0.001), with *P* for interaction being 0.0018. In the PSM 2 cohort, progesterone levels on the trigger day increased by 0.05 ng/mL with an increase in every retrieved oocyte in the LE GnRH-ant group (β 0.05 ng/mL [95% CI 0.04, 0.06], *P* < 0.001), whereas an increase of 0.02 ng/mL was observed in the GnRH-a group (β 0.02 ng/mL [95% CI 0.01, 0.03], *P* < 0.001), with *P* for interaction being 0.0002. LE supplementation on the antagonist protocols may increase progesterone levels in the late follicular stage.

## Introduction

Letrozole (LE) is a drug commonly used to induce ovulation. It is a selective aromatase inhibitor that blocks oestrogen biosynthesis and indirectly increases pituitary secretion of follicle-stimulating hormone (FSH), thus increasing FSH stimulation to the ovary and promoting follicular development (1). Letrozole also enhances intrafollicular androgen levels, which up-regulate and sensitise FSH receptors in the ovary and stimulate activin secretion (2). A meta-analysis confirmed the efficacy and safety of controlled ovarian stimulation (COS) with gonadotropins and LE, considering the number of oocytes retrieved from mature metaphase II, total number of retrieved oocytes, the maturation rate, the fertilisation rate, and the lack of evidence of its harm to the foetus (3, 4).

Using LE during *in vitro* fertilisation and intracytoplasmic sperm injection (IVF/ICSI) treatment has many advantages. First, LE is used to prepare the endometrium in the frozen embryo transfer (FET) cycle, and no negative effect on endometrial receptivity has yet been reported (5–8). Some studies revealed that clinical pregnancy rates and live birth rates in the LE group are significantly higher, and the rate of miscarriage is significantly lower compared to those in the natural and hormone replacement cycle groups (9, 10). Second, LE significantly lowers the level of oestradiol and prevents ovarian hyperstimulation syndrome (11), exerting protective effects in patients with breast cancer (12). Third, adding LE to normal responders can reduce the dosage of gonadotropins, without affecting gravidity (13).

Letrozole competitively binds to the heme group of the cytochrome P450 subunit of aromatase, blocking the conversion of androstenedione and testosterone to oestrone and oestradiol, leading to an increase in androgen and a decrease in oestrogen levels (14). Therefore, LE is commonly used to promote ovulation under conditions of low ovarian reserve to increase the ovarian response to FSH (1, 12). Although high levels of progesterone have been related to using LE during the luteal phase (15, 16), it remains unclear whether LE induces an increase in progestogen during the late follicular phase. In some randomised controlled trials (RCT), LE use in normal responders did not significantly up-regulate progesterone on trigger day (17, 18). Other RCTs showed that LE supplementation increases the incidence of premature progesterone rise in the late follicular phase of IVF/ICSI (19) or increases progesterone levels (14, 20). Reports on the effects of LE on progesterone levels during ovulation induction are inconsistent, largely because the above findings are based on RCTs with small sample sizes. In addition, the progesterone level during ovulation induction was not the main outcome measure in those studies, and the power of test is insufficient. Finally, the different eligibility criteria of the studies limit extrapolations of the results.

The purpose of this study was to determine whether the LE antagonist protocol would increase progesterone levels on the trigger day of ovulation induction in various patients. This study may be useful in improving LE use.

## Materials and Methods

### Study Design and Patients

**Figure 1:**
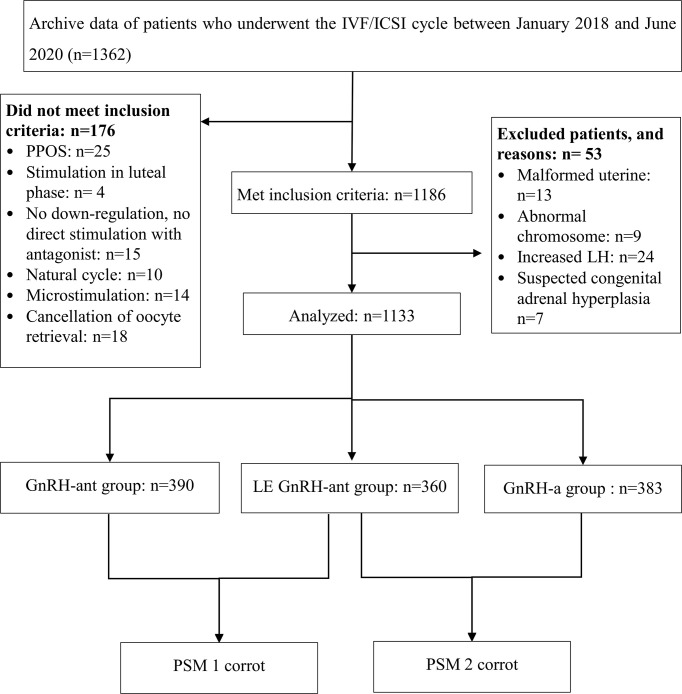
Patient recruitment flowchart. IVF/ICSI: *in vitro* fertilisation or intracytoplasmic sperm injection; GnRH-a: gonadotropin-releasing hormone-agonist; GnRH-ant: gonadotropin-releasing hormone-antagonist; LE: letrozole.

This retrospective cohort study was conducted at the Reproductive Medical Centre, Dongguan SSL Central Hospital, Guangdong, China, and included 1,362 IVF/ICSI cycles between January 2018 and June 2020. All patients were in the first cycle. Among them, 1,186 cycles followed a gonadotropin-releasing hormone-agonist (GnRH-a) or gonadotropin-releasing hormone-antagonist (GnRH-ant) protocol. Females with congenital adrenal hyperplasia, luteinising hormone (LH) levels > 2.5 times the baseline levels on trigger day, a malformed uterus, or an abnormal chromosome were excluded. Based on different ovulation induction protocols, 1,133 infertile women who underwent ovarian stimulation were divided into the following groups: GnRH-a (383), GnRH-ant (390), and LE GnRH-ant (360) (**Figure 1**).

### Data Collection

Age, duration of infertility, body mass index (BMI), anti-Mullerian hormone (AMH) levels during menstruation, factors of infertility, and treatment intervention features (including total gonadotropins, stimulation days, total retrieved oocytes, and hormone levels on trigger day) were collected from the hospital’s medical record system. Patients were followed up routinely until either the occurrence of menstrual periods or childbirth. Follow-ups were censored on 15 April 2021.

Patients on the GnRH-a protocol were administered 0.8–1.5 mg of triptorelin during the mid-luteal phase of the preceding *in vitro* fertilisation-embryo transfer (IVF-ET) cycle. Fourteen days later, the initial dose of 125–300 IU recombinant FSH (rFSH, Gonal F, Merck Serono, Switzerland) was determined based on patient characteristics (age, BMI, AMH, and the antral follicle count).

Patients in the GnRH-ant protocol received daily intramuscular injections of rFSH (125–300 IU) from the first day of stimulation to the day of human chorionic gonadotropin (hCG) administration. When the lead follicle was 14 mm or more in diameter or oestradiol levels were ≥ 400 pg/mL, patients received 0.25 mg of antagonist (Cetrotide; Merk Serono, Switzerland) until they were triggered by hCG (Chorionic Gonadotrophin, Lizhu, China).

Some patients on the GnRH-ant protocol were co-treated with 2.5 mg of LE (Jiangsu, Hengrui, China), which they received daily for four days from stimulation day 1, to reduce costs and increasing ovarian sensitivity.

The administration of FSH was ceased following the appearance of three leading follicles (at least 17 mm in diameter) and 6000–10,000 IU of hCG was subsequently administered. The ovarian response to this therapy was monitored by measuring LH, FSH, serum oestradiol, and progesterone levels and transvaginal ultrasonography. Oocytes were retrieved 35 to 36 h after hCG injection.

### Hormone Level Detection

All hormone levels were measured using a detection kit using electrochemiluminescence (Cobas e411, Roche, Germany). The detection steps were performed under the manufacturer’s instructions. Mean percent intra-assay and inter-assay coefficient of variations, respectively, were 1.8% and 5.2% for LH, 1.8% and 5.3% for FSH, 3.1% and 3.4% for oestradiol, 3.3% and 10.4% for progesterone, 2.1% and 3.2% for testosterone. The low detection limits were 0.100 mIU/mL for LH, 0.100 mIU/mL for FSH, 5 pg/mL for oestradiol, 0.05 ng/mL for progesterone, 0.025 ng/mL for testosterone.

### Statistical Analysis

Propensity score matching (PSM) was performed to balance the factors related to progesterone levels on trigger day. To reduce sample loss, we conducted a pairwise comparison. Propensity score matching was conducted to balance LE GnRH-ant and GnRH-ant groups to form the PSM 1 cohort and another to balance the LE GnRH-ant and GnRH-a groups to form the PSM 2 cohort.

To create comparable groups, the variables and coefficients matched by the two queues were slightly different. Variables in the PSM 1 included age, AMH, BMI, factors of infertility, duration of infertility, total gonadotropins, and total days of GnRH-ant. The matching ratio was 1:1, and the matching range of the score was 0.005. The variables in PSM 2 included age, AMH, BMI, factors of infertility, and duration of infertility. The matching ratio was 1:1, and the matching range of the score was 0.01.

Following PSM to create characteristically similar groups, an interaction analysis was performed on progesterone levels on trigger day change, with the total number of retrieved oocytes increasing in PSM 1 and 2 cohorts.

Premature LH surge may lead to an increase in progesterone and also result in early ovulation. Therefore, we divided LH levels on trigger day into three equal groups (tertile) and compared progesterone levels in each tertile.

Besides progesterone levels on trigger day, four other variables were added to describe the changes in progesterone levels: 1) P1.5, i.e., the proportion of women with a progesterone level of >1.5 ng/ml. Progesterone levels on the trigger day were converted to a binary variable with 1.5 ng/mL as the boundary. Progesterone levels of > 1.5 ng/mL on trigger day can affect the results of embryo transfer, and it is recommended to freeze all embryos before transferring (21). 2) The progesterone to oocyte index (POI), i.e., the relative value of the ratio of progesterone to aspirated oocytes. A high POI predicts poor clinical pregnancy and live birth rates in fresh IVF/ICSI cycles (22). Extreme situations, such as early ovulation, due to which a very few oocytes are obtained on the day of egg retrieval, might have affected the results. Therefore, data on extreme cases (upper and lower 1% of the data distribution) were removed and the remaining data were recorded as POI. 3) The P/E2 ratio, calculated as P (pg/mL)/E2 (pg/mL). 4) P/E2 0.55, i.e., the proportion of women with a P/E2 ratio of > 0.55. Progesterone levels on trigger day were converted into a binary variable, with 0.55 as the boundary. A regression analysis was used to explore the relationship between these four variables and medication regimens.

A student’s t-test was used for normal distribution data, and a Kruskal–Wallis test was used for non-normal distribution data. A chi-square test was used for counting data. If the theoretical number of counting variables was less than 10, a Fisher’s exact test was used. All analyses were performed using Empower (R) (X&Y Solutions, Inc, Boston, MA, USA) and R software (version 3.3.3, The R Foundation for Statistical Computing, Vienna, Austria). Statistical significance was established at *P* < 0.05.

## Results

Before PSM, patients in the LE GnRH-ant group were older (*P* < 0.001) and had a higher BMI (*P* = 0.008) and a frequency of ovulation disorder and male factor (*P* < 0.001) than those in the other two groups (**Supplemental Table 1**). **Supplemental Table 2** presents the results of ovulation induction before PSM.

After PSM, 266 patients in the GnRH-ant group were successfully matched to 266 patients in the LE GnRH-ant group (PSM 1 cohort, **Table 1**). There was no statistically significant difference in patient characteristics between the two groups; however, there were significant differences in treatment results between the two groups (PSM 1 cohort, **Table 2**). Patients in the LE GnRH-a group presented higher progesterone levels (1.22 ± 0.95 ng/mL vs 0.86 ± 0.60 ng/mL, *P* < 0.001), with higher proportion of patients with a progesterone level > 1.5 ng/ml (24.81% vs 7.52%, *P* < 0.001); higher P/E2 ratio (0.75 ± 0.58 vs 0.43 ± 0.60, *P* < 0.001), with higher proportion of patients with a P/E2 ratio > 0.55 (52.63% vs 17.67%, *P* < 0.001); higher POI (132.47 ± 112.07 pg/mL vs 100.84 ± 93.87 pg/mL, *P* < 0.001), and lower oestradiol levels on trigger day (2,259.90 ± 1,779.14 pg/mL vs 2,773.54 ± 1,830.26 pg/mL, *P* < 0.001) than those in the GnRH-ant group.

**Table 1:** Baseline characteristics of patients and the features of ovulation induction after propensity score matching.

Variables	PSM 1 cohort			PSM 2 cohort		
	GnRH-ant	LE GnRH-ant	*P* value	GnRH-a	LE GnRH-ant	*P* value
	(n = 266)	(n = 266)		(n = 283)	(n = 283)	
Duration of infertility (years)	3.85 ± 3.14	3.77 ± 2.90	0.785	3.86 ± 3.06	4.08 ± 3.23	0.401
Age (years)	32.62 ± 5.60	32.47 ± 5.36	0.758	32.00 ± 4.69	32.18 ± 5.05	0.648
BMI (kg/m^2^)	21.93 ± 3.01	21.99 ± 3.10	0.826	21.86 ± 3.08	22.06 ± 3.01	0.448
AMH (ng/mL)	3.72 ± 2.73	3.79 ± 2.93	0.802	4.64 ± 3.15	4.59 ± 3.80	0.862
Basic FSH (mIU/mL)	7.99 ± 3.92	7.65 ± 3.51	0.301	7.11 ± 1.98	7.42 ± 3.27	0.178
Basic E2 (pg/mL)	45.31 ± 35.35	49.15 ± 54.49	0.336	44.19 ± 21.36	48.95 ± 53.58	0.166
Basic P (ng/mL)	0.40 ± 0.94	0.49 ± 1.31	0.322	0.44 ± 1.84	0.53 ± 1.76	0.583
Basic LH (mIU/mL)	6.04 ± 3.80	5.71 ± 3.08	0.273	6.19 ± 3.10	5.90 ± 3.34	0.289
Basic T (ng/mL)	0.27 ± 0.18	0.45 ± 2.87	0.332	0.47 ± 3.21	0.45 ± 2.78	0.937
Factors of infertility			0.190			0.152
Ovulation disorder	32 (12)	44 (16.5)		51 (18)	63 (22.3)	
Tubal factors	164 (61.7)	142 (53.4)		167 (59)	145 (51.2)	
Endometriosis	6 (2.3)	13 (4.9)		8 (2.8)	13 (4.6)	
Male factors	27 (10.2)	28 (10.5)		16 (5.7)	26 (9.2)	
Other	37 (13.9)	39 (14.7)		41 (14.5)	36 (12.7)	
Total gonadotropins (IU)	1,951.64 ± 714.07	1,945.63 ± 675.96	0.921	2,664.80 ± 910.41	1,988.19 ± 755.86	< 0.001
Days of stimulation	9.49 ± 1.94	9.65 ± 2.05	0.374	12.51 ± 2.18	9.72 ± 2.05	< 0.001
Total GnRH-ants (mg)	1.35 ± 0.47	1.37 ± 0.49	0.726	/	/	

GnRH-ant: gonadotropin-releasing hormone-antagonist; LE GnRH-ant: GnRH-ant + letrozole; BMI: body mass index; AMH: anti-Mullerian hormone; LH: Luteinizing hormone; FSH: follicle-stimulating hormone; P: progesterone; E2: oestradiol.

**Table 2:** Results of ovulation induction after propensity score matching.

Variables	PSM 1 cohort			PSM 2 cohort		
	GnRH-ant	LE GnRH-ant	*P* value	GnRH-a	LE GnRH-ant	*P* value
	(n = 266)	(n = 266)		(n = 283)	(n = 283)	
Total number of retrieved oocytes	12.16 ± 7.99	12.26 ± 8.39	0.889	16.78 ± 8.47	13.30 ± 8.56	<0.001
Estradiol levels on trigger day (pg/mL)	2,773.54 ± 1,830.26	2,259.90 ± 1,779.14	0.001	3,658.56 ± 1972.96	2,347.71 ± 1,781.67	<0.001
LH levels on trigger day (mIU/mL)	3.62 ± 3.07	5.26 ± 5.48	<0.001	1.20 ± 0.91	5.07 ± 4.50	<0.001
P levels on trigger day (ng/mL)	0.86 ± 0.60	1.22 ± 0.95	<0.001	0.98 ± 0.61	1.23 ± 0.91	<0.001
PE2	0.43 ± 0.60	0.75 ± 0.58	<0.001	0.32 ± 0.21	0.72 ± 0.53	<0.001
POI (pg/mL)	100.84 ± 93.87	132.47 ± 112.07	<0.001	74.37 ± 63.25	118.54 ± 90.06	<0.001
P1.5			<0.001			<0.001
≤1.5 (ng/mL)	246 (92.48%)	200 (75.19%)		247 (87.30%)	211 (74.55%)	
>1.5 (ng/mL)	20 (7.52%)	66 (24.81%)		36 (12.70%)	72 (25.45%)	
PE2 0.55			<0.001			<0.001
≤0.55	219 (82.33%)	126 (47.37%)		251 (88.69%)	136 (48.06%)	
>0.55	47 (17.67%)	140 (52.63%)		32 (11.31%)	147 (51.94%)	

GnRH-a, gonadotropin-releasing hormone-agonist; GnRH-ant, gonadotropin-releasing hormone-antagonist; LE GnRH-ant, GnRH-ant + letrozole; LH, Luteinizing hormone; P, progesterone; E2, oestradiol; P1.5, the proportion of women with a progesterone level > 1.5 ng/mL; POI, relative value of progesterone to an aspirated oocytes ratio; P/E2 0.55, the proportion of women with a P/E2 ratio > 0.55.

In the PSM 2 cohort, 283 patients in the GnRH-a group were successfully matched to 283 patients in the LE GnRH-ant group (PSM 2 cohort, **Table 1**). There was no statistically significant difference in patient characteristics between the two groups. In the LE GnRH-ant group, the total Gn used was less than that of the GnRH-a group (1988.19 ± 755.86 IU vs 2,664.80 ± 910.41 IU, *P* < 0.001), and the total stimulation days were also relatively less than those in the GnRH-a group (9.72 ± 2.05 days vs 12.51 ± 2.18 days, *P* < 0.001). However, there were significant differences in treatment results between the two groups (PSM 2 cohort, **Table 2**). Patients in the LE GnRH-a group presented higher progesterone levels on trigger day (1.23 ± 0.91 ng/mL vs 0.98 ± 0.61 ng/mL, *P* < 0.001), with higher proportion of patients with a progesterone level > 1.5 ng/ml (25.45% vs 12.70%, *P* < 0.001); higher P/E2 ratio (0.72 ± 0.53 vs 0.32 ± 0.21, *P* < 0.001), with higher proportion of patients with a P/E2 ratio > 0.55 (51.94% vs 11.31%, *P* < 0.001); higher POI (131.23 ± 109.62 pg/mL vs 97.28 ± 89.77 pg/mL, *P* < 0.001), lower oestradiol levels on trigger day (2,347.71 ± 1,781.67 pg/mL vs 3,658.56 ± 1972.96 pg/mL, *P* < 0.001), and less retrieved oocytes (13.30 ± 8.56 vs 16.78 ± 8.47, *P* < 0.001) than those in the GnRH-a group.

In the PSM 1 cohort, to determine whether high level of LH in the LE GnRH-ant protocol is caused due to high progesterone levels, LH was divided into three groups (tertiles). The progesterone level, proportion of patients with a progesterone level > 1.5 ng/mL, P/E2 ratio, and proportion of patients with a P/E2 ratio > 0.55 in the LE GnRH-ant group were significantly higher than those in the GnRH-ant group in each subgroup analysis (**Supplemental Table 3**).

**Figure 2:**
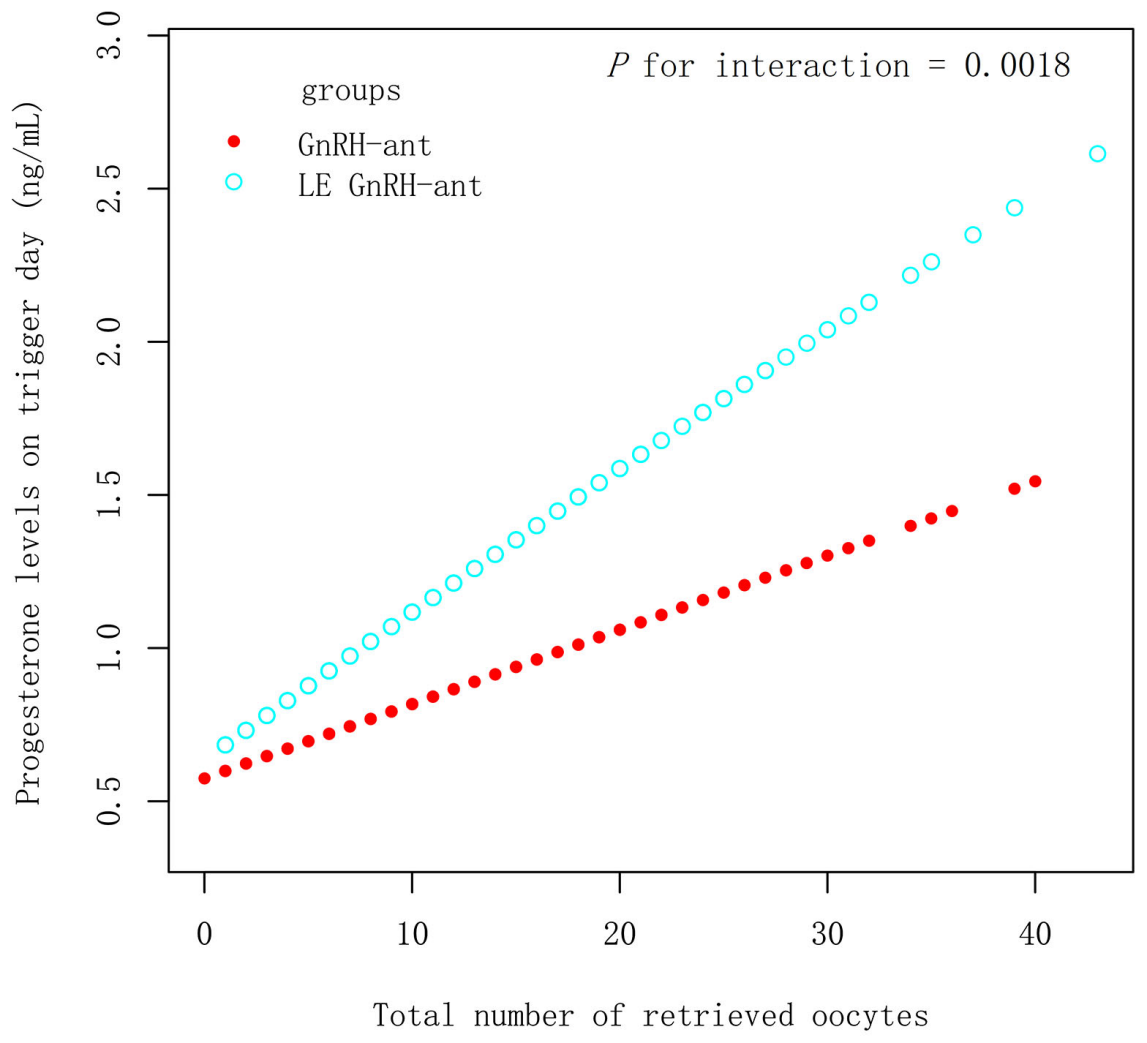
Relationship between progesterone levels and the number of retrieved oocytes in GnRH-ant and LE GnRH-ant groups. GnRH-a: gonadotropin-releasing hormone-agonist; GnRH-ant: gonadotropin-releasing hormone-antagonist; P1.5: the proportion of women with a progesterone level >1.5 ng/mL.

**Figure 3:**
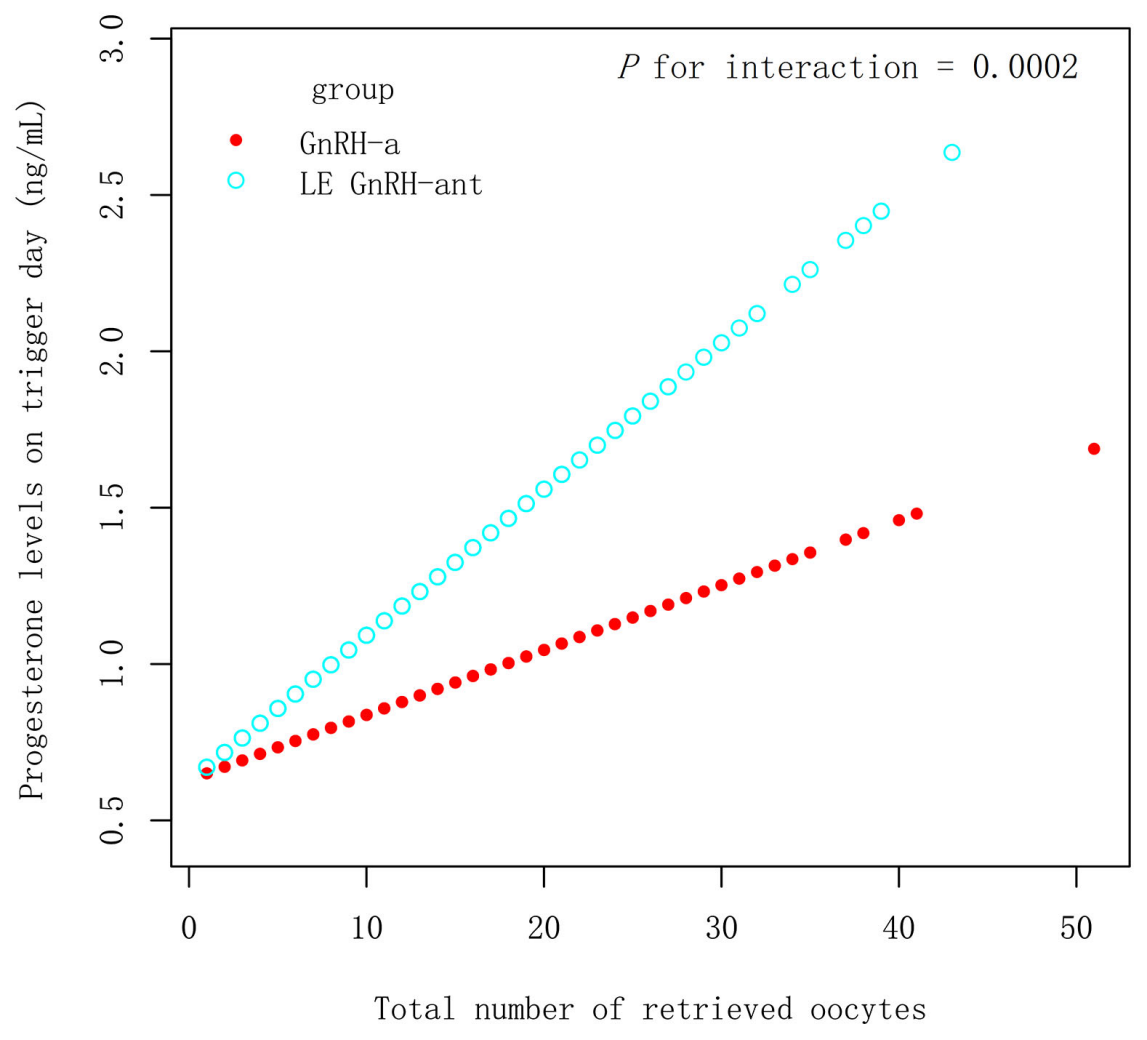
Relationship between progesterone levels and the number of retrieved oocytes in GnRH-a and LE GnRH-ant groups. GnRH-a: gonadotropin-releasing hormone-agonist; GnRH-ant: gonadotropin-releasing hormone-antagonist; P1.5: the proportion of women.

In the PSM 1 cohort, progesterone levels on the trigger day increased by 0.05 ng/mL with an increase in all retrieved oocyte in the LE GnRH-ant group (β 0.05 ng/mL [95% CI 0.04, 0.06], *P* < 0.001), whereas an increase of 0.02 ng/mL was observed in the GnRH-ant group (β 0.02 ng/mL [95% CI 0.01, 0.03], *P* < 0.001) (**Figure 2**), with *P* for interaction being 0.0018, which suggest, with increased retrieved oocytes, women in the LE GnRH-ant group had a greater increase in progesterone levels than women in the GnRH-ant group. In the PSM 2 cohort, progesterone levels on the trigger day increased by 0.05 ng/mL with an increase in every retrieved oocytes in the LE GnRH-ant group (β 0.05 ng/mL [95% CI 0.04, 0.06], *P* < 0.001), whereas an increase of 0.02 ng/mL was observed in the GnRH-a group (β 0.02 ng/mL [95% CI 0.01, 0.03], *P* < 0.001) (**Figure 3**), with *P* for interaction was 0.0002, which suggest, with increased retrieved oocytes, women in the LE GnRH-ant group had a greater increase in progesterone levels than women in the GnRH-a group.

## Discussion

This retrospective matched cohort study explored the influence of adopting antagonist protocols that introduced LE during ovulation stimulation on trigger day on the hormone levels in infertile females undergoing IVF/ICSI. The results indicated that the antagonist protocols that introduced LE increased the risk of high progesterone levels on trigger day.

Many studies have shown that progesterone produced in IVF/ICSI is related to the number of follicles (23, 24). In this study, we hypothesised that late elevations in follicular blood progesterone might be owing to the following reasons: 1) An increase in progesterone secretion from the recruited follicles; the greater the number of follicles, the higher the progesterone level, which could be induced by any protocol; 2) pathological progestogen, which is related to the premature luteinisation of follicles and decreased follicle quality. This increase is always accompanied with LH surge; and 3) use of LE.

In the PSM 1 cohort, increased progesterone levels strongly point to LE use. First, progesterone levels are much higher in the LE GnRH-ant group under the conditions where the number of eggs was similar (**Table 2**). Second, **Figure 2** provides evidence that with increased eggs, women in the LE GnRH-ant group had a greater increase in progesterone levels than women in the GnRH-ant group. These evidences eliminate the possibility that the increased progesterone levels in the LE GnRH-ant group are attributed to the increase in the number of eggs. Third, there is no association between the increase in LH and progesterone levels according to the tertiles stratified analysis. Progesterone in the LE GnRH-ant group increased by 0.40 ng/mL even if LH levels were comparatively lower (≤2.39 mIU/mL) than those in the GnRH-ant group (**Supplemental Table 3**). Simultaneously, the proportion of patients with a progesterone level >1.5 ng/mL was 3.70 times higher in the LE GnRH-ant group.

Similarly, the progesterone levels of the late follicles were also higher in the LE GnRH-ant group than in the GnRH-a group in the PSM 2 cohort. **Figure 3** provides evidence that the protocol is an effect modifier that affects progesterone levels and the number of eggs in the PSM 2 cohort. Progesterone levels are relatively easier to increase with the increase in the number of eggs in the LE GnRH-ant group.

Our research indicates that LE poses a strong risk of increasing progesterone levels. Regarding the mechanism through which LE elevates progesterone, we hypothesised LE inhibited the production of oestrogen (mainly oestradiol), resulting in the accumulation of oestrogen synthesis precursors and androgens (e.g., testosterone and androstenedione) (14). Once the accumulation of androgens reaches a certain threshold, progesterone accumulates, increasing its levels in the blood, which has also been proven in previous studies (14, 19).

High progesterone levels have a negative impact on the pregnancy rate on the trigger day of the fresh cycle. Studies have demonstrated that an increase in progesterone during the late follicular phase is associated with decreased pregnancy potential (25). A cut-off level of 1.5 ng/mL appeared to discriminate between those with and without negative pregnancy outcomes (18, 19). The decreased endometrial receptivity owing to an early increase in progesterone levels may explain the decrease in pregnancy rates (26).

Other studies have shown that progesterone level has a marked negative effect in the group where < 5 oocytes are retrieved; however, this is not observed among high responders (27). Some researchers believe that the number of mature follicles is very important in the evaluation of progesterone, so POI and P/E2 ratio were offered to assess the effects of progesterone. The POI values greater than 0.36 ng/mL/oocyte result in a low clinical pregnancy rate and a live birth rate of 8.0% and 5.9%, respectively (28). Shufaro et al. reported that the POI is inversely related to pregnancy rate and concluded that the POI is better correlated with IVF outcomes than blood progesterone levels (29). Besides POI, the P/E2 ratio was proposed as important when considering the number of developing follicles in COS cycles. A P/E2 ratio > 0.55 affects the clinical pregnancy rate, and the P/E2 ratio is the only independent prognostic factor for cycle outcomes in females undergoing cleavage-stage embryo transfer (30). These findings emphasise the complexity of analysing data on end-COS progesterone. In this study, these indicators were considered to evaluate the potential effects of LE. The indicators were upregulated in the LE GnRH-ant group. However, in this study, we can only prove the elevated progesterone associated with LE, the effects of LE on pregnancy rate and live birth rate need to be further analysed.

In contrast to previous studies (17–20), this study included a large sample size with high, normal, and low response groups, resulting in different eggs obtained after appropriate stimulation. **Figure 2** and **Figure 3** show that with increased eggs, women in the LE GnRH-ant group had a greater increase in progesterone levels than women in the GnRH-ant group or the GnRH-a group. Consequently, fewer retrieved oocytes result in a slight increase in progesterone levels, and therefore a slight difference is observed between the groups. We speculated that when there are fewer eggs, the precursor of oestrogen synthesis of androgen accumulation caused by LE is less, and progesterone accumulation is even lower, which makes it difficult to find a significant difference in LE arms. This may explain the cause of rising testosterone levels in parts of the follicular fluid, without detecting the increase in progesterone, in LE-related studies, with poor responders (31). Meanwhile, an increase in the number of follicles means an increase in the accumulation of precursors in the presence of LE. A study has reported that in patients with high responses (the average number of retrieved oocytes is 12–13), the progesterone levels significantly increase and the proportion of patients with a progesterone level >1.5 ng/mL also increases significantly (19). In patients with normal responses (the average number of retrieved oocytes is 7.7–8.0), researchers reported that progesterone levels show a significant increase; however, no increase in the proportion of women with a progesterone level >1.5 ng/mL is observed (14). A small sample size may also be responsible for no difference in the proportion of patients with a progesterone level >1.5 ng/mL in some studies.

This study is a retrospective observational study, with an inherent challenge of bias and/or confounders. PSM was efficient in minimising bias due to confounding. PSM matched two groups of study participants with similar or identical propensity scores, to create samples approximate to a randomised trial by directly comparing outcomes between individuals. PSM can improve estimation of the causal treatment effect in an observational study by mimicking some of the statistical properties of a randomised controlled trial (32). Therefore, we chose PSM instead of regression analyses for the confounders in this study.

The current study had limitations. First, the detection of serum progesterone levels was based on results obtained by chemiluminescence, but not mass spectrometry, which may have affected their accuracy. Second, there were some unmeasurable confounders in this single-centre retrospective PSM cohort study, and this limitation can be overcome by RCT studies with sufficient power of test. Third, changes in serum progesterone-androgen-oestrogen metabolism were not detected to confirm the source of the rising progesterone. These issues should be addressed in future research.

In conclusion, the GnRH-ant protocols that utilise LE increase the risk of higher progesterone levels, P/E2 ratio, POI, the proportion of females with a P/E2 ratio >0.55, as well as a higher proportion of those with a progesterone level >1.5 ng/mL on trigger day. These results suggest LE should be handled with caution during ovarian stimulation for IVF/ICSI and that a greater focus should be on high serum progesterone levels. However, these results still need to be further confirmed by a well-designed RCT study with sufficient power of test.

## Data Availability Statement

The raw data supporting the conclusions of this article will be made available by the authors, without undue reservation.

## Ethics Statement

The study protocol was approved by the Institutional Ethics Committee of Dongguan Songshan Lake (SSL) Central Hospital (202005010). The requirement for informed consent was waived because of the retrospective nature of the study.

## Author Contributions

RL: Conceptualisation, methodology, formal analysis, writing- original draft preparation, writing- reviewing and editing, project administration, funding acquisition. LZ: Investigation, data curation, writing- reviewing and editing. XC: Resources, investigation. HH: Investigation, writing- reviewing and editing. ZC: Resources, investigation. All authors have read and contributed to the manuscript. All authors contributed to the article and approved the submitted version.

## Funding

This research was funded by the talent introduction program initiated by Dongguan SSL Central Hospital (#YJ-20190001), and the key project of social science and technology development of Dongguan City in 2019 (201950715024177).

## Conflict of Interest

The authors declare that the research was conducted in the absence of any commercial or financial relationships that could be construed as a potential conflict of interest.

## Publisher’s Note

All claims expressed in this article are solely those of the authors and do not necessarily represent those of their affiliated organizations, or those of the publisher, the editors and the reviewers. Any product that may be evaluated in this article, or claim that may be made by its manufacturer, is not guaranteed or endorsed by the publisher.
