# Supplementary material for: Letrozole Supplementation and the Increased Risk of Elevated Progesterone Levels on Trigger Day
Source: Front Endocrinol (Lausanne). 2022 Jul 25;13:904089. doi: 10.3389/fendo.2022.904089 (PMC9359123; doi:10.3389/fendo.2022.904089)
Supplement: Supplementary file 1 [file DataSheet_1.docx]

Supplementary Material

# Supplementary Tables

**Supplemental Table 1**. Baseline characteristics of patients and the features of ovulation induction before propensity score matching.

| Characteristic | LE GnRH-ant | GnRH-a | GnRH-ant | *P* value |
| --- | --- | --- | --- | --- |
|  | (n = 360) | (n = 383) | (n = 390) |  |
| Age (years) | 33.16 ± 5.59 | 31.48 ± 4.73 | 32.33 ± 5.54 | < 0.001 |
| BMI (kg/m^2^) | 22.23 ± 3.08 | 21.66 ± 3.03 | 21.60 ± 2.98 | 0.008 |
| Duration of infertility (years) | 4.11 ± 3.44 | 3.93 ± 3.01 | 3.64 ± 3.00 | 0.125 |
| AMH (ng/mL) | 4.22 ± 3.64 | 4.54 ± 3.13 | 3.56 ± 2.64 | < 0.001 |
| Basic LH (mIU/mL) | 5.83 ± 3.29 | 6.22 ± 3.15 | 5.88 ± 3.47 | 0.215 |
| Basic FSH (mIU/mL) | 7.62 ± 3.46 | 7.07 ± 1.99 | 8.16 ± 4.23 | < 0.001 |
| Basic P (ng/mL) | 0.57 ± 1.79 | 0.43 ± 1.60 | 0.65 ± 3.73 | 0.495 |
| Factors of infertility |  |  |  | < 0.001 |
| Ovulation disorder | 75 (20.83%) | 63 (16.45%) | 43 (11.03%) |  |
| Tubal factors | 185 (51.39%) | 216 (56.40%) | 241 (61.79%) |  |
| Endometriosis | 15 (4.17%) | 10 (2.61%) | 11 (2.82%) |  |
| Male factors | 39 (10.83%) | 18 (4.70%) | 41 (10.51%) |  |
| Other | 46 (12.78%) | 76 (19.84%) | 54 (13.85%) |  |
| Total gonadotropins (IU) | 2,024.57 ± 730.63 | 2,631.36 ± 872.45 | 1,876.03 ± 691.76 | < 0.001 |
| Days of stimulation | 9.65 ± 2.05 | 12.45 ± 2.07 | 9.41 ± 2.06 | < 0.001 |
| Total GnRH-ants (mg) | 1.31 ± 0.48 | / | 1.39 ± 0.65 | < 0.001 |

GnRH-ant: gonadotropin-releasing hormone antagonist; LE GnRH-ant: GnRH-ant + letrozole; BMI: body mass index; AMH: anti-Mullerian hormone; LH: Luteinizing hormone; FSH: follicle-stimulating hormone; P: progesterone; E2: oestradiol.

**Supplemental Table 2**. Results of ovulation induction before propensity score matching.

| Characteristic | LE GnRH-ant | GnRH-a | GnRH-ant | *P* value |
| --- | --- | --- | --- | --- |
|  | (n = 360) | (n = 383) | (n = 390) |  |
| Total number of retrieved oocytes | 12.39 ± 8.49 | 16.68 ± 8.28 | 12.11 ± 7.85 | < 0.001 |
| Estradiol levels on trigger day (pg/mL) | 2,201.85 ± 1,736.63 | 3,654.83 ± 1930.89 | 2,785.99 ± 1,822.39 | < 0.001 |
| LH levels on trigger day (mIU/mL) | 5.37 ± 5.50 | 1.27 ± 1.10 | 3.61 ± 2.90 | < 0.001 |
| P levels on trigger day (ng/mL) | 1.19 ± 0.88 | 0.96 ± 0.62 | 0.85 ± 0.55 | < 0.001 |
| P1.5 |  |  |  | < 0.001 |
| ≤1.5 (ng/mL) | 271 (75.28%) | 333 (86.95%) | 360 (92.31%) |  |
| >1.5 (ng/mL) | 89 (24.72%) | 50 (13.05%) | 30 (7.69%) |  |
| POI (pg/mL) | 128.54 ± 106.52 | 71.16 ± 58.27 | 98.43 ± 87.62 | < 0.001 |
| P/E2 | 0.75 ± 0.56 | 0.31 ± 0.23 | 0.43 ± 0.53 | < 0.001 |
| P/E2 0.55 |  |  |  | < 0.001 |
| ≤0.55 | 164 (45.56%) | 343 (89.56%) | 320 (82.05%) |  |
| >0.55 | 196 (54.44%) | 40 (10.44%) | 70 (17.95%) |  |

GnRH-a: gonadotropin-releasing hormone agonist; GnRH-ant: gonadotropin-releasing hormone antagonist; LE GnRH-ant: GnRH-ant + letrozole; LH: Luteinizing hormone; P: progesterone; E2: oestradiol; P1.5: the proportion of women with a progesterone level >1.5 ng/mL; POI: relative value of progesterone to an aspirated oocytes ratio; P/E2 0.55: the proportion of women with a P/E2 ratio >0.55.

**Supplemental Table 3.** Differences in progesterone levels between the LE antagonist and antagonist groups by LH quartiles categorisation.

| LH tertile | ≤ 2.39 (mIU/mL) | | 2.4–4.5 (mIU/mL) | | > 4.5 (mIU/mL) | | Total | |
| --- | --- | --- | --- | --- | --- | --- | --- | --- |
|  | β (OR) (95%CI) | *P* | β (OR) (95%CI) | *P* | β (OR) (95%CI) | *P* | β (OR) (95%CI) | *P* |
| P levels on trigger day (ng/mL) | |  |  |  |  |  |  |  |
| GnRH-ant | 0 |  | 0 |  | 0 |  | 0 |  |
| LE GnRH-ant | 0.40 (0.17, 0.64) | 0.001 | 0.34 (0.06, 0.62) | 0.018 | 0.46 (0.28, 0.64) | < 0.001 | 0.40 (0.26, 0.54) | < 0.001 |
| PE2 |  |  |  |  |  |  |  |  |
| GnRH-ant | 0 |  | 0 |  | 0 |  | 0 |  |
| LE GnRH-ant | 0.29 (0.18, 0.41) | < 0.001 | 0.20 (-0.02, 0.41) | 0.080 | 0.38 (0.20, 0.56) | < 0.001 | 0.29 (0.19, 0.39) | < 0.001 |
| POI (pg/mL) |  |  |  |  |  |  |  |  |
| GnRH-ant | 0 |  | 0 |  | 0 |  | 0 |  |
| LE GnRH-ant | 19.82 (-0.26, 39.90) | 0.055 | -4.86 (-32.62, 22.90) | 0.732 | 49.95 (8.52, 91.38) | 0.019 | 20.52 (2.71, 38.33) | 0.024 |
| P1.5 |  |  |  |  |  |  |  |  |
| GnRH-ant | 1 |  | 1 |  | 1 |  | 1 |  |
| LE GnRH-ant | 3.70 (1.75, 7.83) | < 0.001 | 4.47 (1.57, 12.72) | 0.005 | 17.70 (2.34, 133.95) | 0.005 | 4.87 (2.79, 8.51) | < 0.001 |
| PE2 0.55 |  |  |  |  |  |  |  |  |
| GnRH-ant | 1 |  | 1 |  | 1 |  | 1 |  |
| LE GnRH-ant | 5.00 (2.31, 10.85) | < 0.001 | 4.81 (2.42, 9.57) | < 0.001 | 4.06 (2.10, 7.84) | < 0.001 | 4.56 (3.05, 6.84) | < 0.001 |

GnRH-ant: gonadotropin-releasing hormone antagonist; LE GnRH-ant: GnRH-ant + letrozole; LH: Luteinizing hormone; FSH: follicle-stimulating hormone; P: progesterone; E2: oestradiol; P1.5: the proportion of women with a progesterone level >1.5 ng/mL; POI: relative value of progesterone to an aspirated oocytes ratio; P/E2 0.55: the proportion of women with a P/E2 ratio >0.55.
